# The relationship between quality of sleep and night shift rotation interval

**DOI:** 10.1186/s40557-015-0084-x

**Published:** 2015-12-17

**Authors:** Jae Youn Kim, Chang Ho Chae, Young Ouk Kim, Jun Seok Son, Ja Hyun Kim, Chan Woo Kim, Hyoung Ouk Park, Jun Ho Lee, Sun Il Kwon

**Affiliations:** Department of Occupational & Environmental Medicine, Samsung Changwon Hospital, School of Medicine, Sungkyunkwan University, 158, Paryong-ro, Changwon-si, 51353 Gyeongsangnam-do Korea (Republic)

## Abstract

**Background:**

Shift work is closely related with workers' health. In particular, sleep is thought to be affected by shift work. In addition, shift work has been reported to be associated with the type or direction of shift rotation, number of consecutive night shifts, and number of off-duty days. We aimed to analyze the association between the night shift rotation interval and the quality of sleep reported by Korean female shift workers.

**Methods:**

In total, 2,818 female shift workers from the manufacturing industry who received an employee physical examination at a single university hospital from January to August in 2014 were included. Subjects were classified into three groups (A, B, and C) by their night shift rotation interval. The quality of sleep was measured using the Korean version of the Pittsburgh Sleep Quality Index (PSQI). Descriptive analysis, univariate logistic regression, and multivariate logistic regression were performed.

**Results:**

With group A as the reference, the odds ratio (OR) for having a seriously low quality of sleep was 1.456 (95% CI 1.171–1.811) and 2.348 (95% CI 1.852–2.977) for groups B and C, respectively. Thus, group C with the shortest night shift rotation interval was most likely to have a low quality of sleep. After adjustment for age, obesity, smoking status, alcohol consumption, exercise, being allowed to sleep during night shifts, work experience, and shift work experience, groups B and C had ORs of 1.419 (95% CI 1.134–1.777) and 2.238 (95% CI 1.737–2.882), respectively, compared to group A.

**Conclusion:**

Our data suggest that a shorter night shift rotation interval does not provide enough recovery time to adjust the circadian rhythm, resulting in a low quality of sleep. Because shift work is influenced by many different factors, future studies should aim to determine the most optimal shift work model and collect accurate, prospective data.

## Introduction

With the advancement of various industries, different types of shift work are being implemented throughout the world. Although the definition of shift work has not been made clear in the literature, most studies have referred to shift work as hours worked during and outside of regular weekdays that are organized into 2-shift or 3-shift systems and include night work, rotation work, and work during irregular hours [1]. Globally, approximately 15–30 % of employees do shift work [2, 3]. In South Korea, a report by Statistics Korea in 2011 on 3414 companies with ten or more employees reported that approximately 15.2 % of these companies adopted shift work systems [4].

The association between shift work and health has been thoroughly investigated through large cohort studies, such as the Nurses’ Health Study from the US [5]. Moreover, studies have reported that numerous factors are related with shift work, including physiological mechanisms such as the inappropriate secretion of melatonin [6] or cortisol [7], a reduced activity of the sympathetic nerve system [8], sleep disorders [9], cardiovascular diseases [10, 11], metabolic syndrome [12], gastrointestinal diseases [13], psychiatric disorders [14], anxiety [15], or stress [16]. In addition, tumors such as breast cancer [17], colorectal cancer [18], and prostatic cancer [19] are known to be related to shift work.

In addition, numbers of studies have been performed to establish relationship between shift work and sleep [20–24]. One of the causes of sleep disorders due to shift work is the disruption of the circadian rhythm [20,25]. The circadian rhythm controls the diurnal variation of the sleep-wake cycles as well as various physiological functions [26]. Although the circadian rhythm cannot be directly measured, problems in the rhythm can be identified by identifying changes in these physiological functions. In fact, previous reports have found that the secretion of melatonin [6], the diurnal rhythm of cortisol [7], and the regulation of core body temperature [27], all of which are closely associated with sleep, are not present in shift workers. As shift work is not a temporary environmental change, like jet lag, chronic disruptions in the circadian clock can result. Therefore, shift work should be regarded as a highly important cause of circadian rhythm disruption.

In general, it reportedly takes approximately one hour of correction for every 24 h of change to a circadian rhythm [28]. In this sense, the night shift rotation interval(times between successive night shift days group and next successive night shift days group) is the recovery period for these disrupted circadian rhythms, therefore, is a crucial factor in studying shift work sleep disorders. But there are no direct associated studies about night shift rotation interval.

Some previous studies have reported that the quality of sleep in employees did not depend on what kind of shift system they worked (a 3-shift system or 2-shift system) [29, 30]. Because a 3-shift system has a longer night shift rotation interval than a 2-shift system does, the results of those studies may mean a difference of sleep quality by night shift rotation interval may be not large. However, these previous studies did not control for the direction of shift or number of consecutive night shifts. Therefore, it is difficult to understand the actual changes to night shift rotation interval and their impact on sleep quality. In addition, the subjects from previous studies had up to three to four days per week as off-duty days. This work schedule is very uncommon in South Korea.

Previous findings have also suggested that women shift workers have a lower quality of sleep than men shift workers do [31]. Furthermore, studies on female workers other than nurses are rare. To this end, we aimed to study the influence of night shift rotation interval on sleep quality in South Korean women workers in the manufacturing industry.

## Methods

### Subjects

**Fig. 1:**
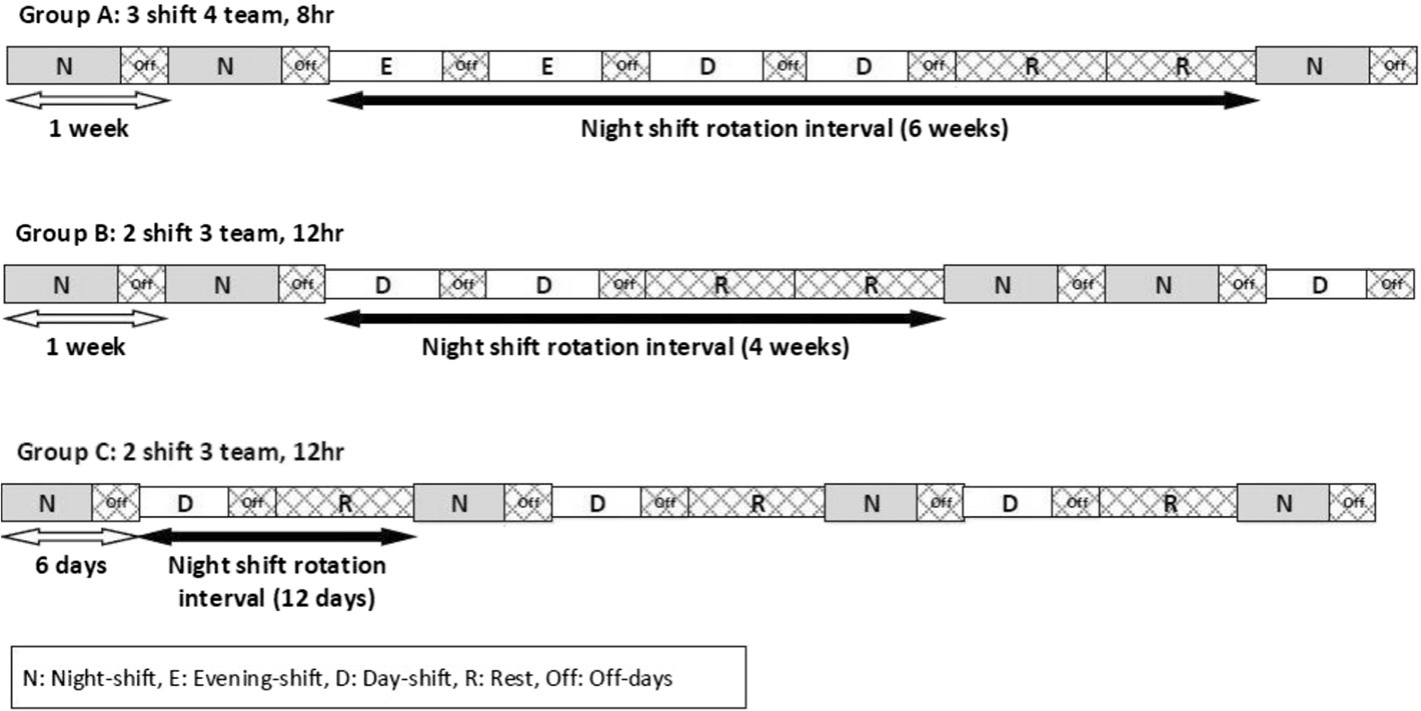
Schematic diagram of shift rotation interval

In total, 2877 female shift workers who received an employee physical examination between January to August 2014 at a single university hospital were eligible for our study. Among them, 54 subjects who did not complete the questionnaire and five with missing data were excluded. Finally, 2818 females were included in this study. The subjects were categorized into one of three groups (A, B, or C) according to their night shift rotation interval. Those in groups A and B worked in the same electronic appliance manufacturing plant. Group A’s shifts changed every two weeks based on the schedule for their 4-team 3-shift system. Group B worked in the 3-team 2-shift system and rotated shifts every two weeks. Those in group C worked at a different electronic appliance manufacturing plant and had the same 3-team 2-shift system as group B, but group C’s shift changed every six days. Figure 1 shows a schematic diagram of shift schedule and night shift rotation interval. Group A had the longest interval, 6 weeks, Group B had 4 weeks, and Group C had the shortest interval, 12 days. At the point of consecutive working times, all groups had more than those of 3 days, so that means they had worked with slow rotating shift.

This study was reviewed by the institutional review board (IRB) before implementation and all questionnaires were obtained under consent of the participants (IRB No. 2015-SCMC-005-00).

### Study variables and measurements

#### General characteristics

Demographic and life style characteristics including age, marital status, education level, body mass index, alcohol consumption, smoking status, exercise, work experience, and shift work experience were investigated. The age range was 20–39 years, and participants were divided into two age groups: 20–29 and 30–39 years. Marital status was classified as unmarried, married, or divorced; there were no widowed subjects. The highest level of education completed was collected as graduating from middle school, high school, junior college, a 4-year university, or a graduate program. These data were further categorized as high school and below or junior college and above. Body mass index was used to classify subjects as underweight (<18.5 kg/m^2^), normal weight (18.5–24.9 kg/m^2^), or overweight (≥25.0 kg/m^2^), according to the World Health Organization criteria [32].

Alcohol consumption was measured by calculating the average amount of alcohol consumed per week in grams, and then subjects were classified as non-drinkers, moderate drinkers (<60 g/week), or heavy drinkers (≥60 g/week). In addition, subjects were classified as non-smokers or smokers. Exercise was classified as exercising <3 times per week or ≥3 times per week. Sleep occurring during work hours was also investigated. Last the length of work experience and shift work experience were categorized as <5 years, 5–9 years, or ≥10 years for each variable.

#### Sleep quality

We used the Korean version of the Pittsburgh Sleep Quality Index (PSQI) [33]. The PSQI consists of seven components: subjective sleep quality, sleep latency, sleep duration, habitual sleep efficiency, sleep disturbances, use of sleep medication, and daytime dysfunction. Scoring of answers is based on a 0 to 3 scale, whereby 3 reflects the negative extreme on the Likert scale (0 = not at all, 3 = extremely). The total possible score is 21 points. We categorized subjects as those with good sleep quality (≤5 points) or bad sleep quality (≥6 points) based on the recommendations made by Buysee et al. [34]. Of the detailed items that make up the quality of sleep in PSQI, to identify items shown in the differences between the three groups, the answers were divided into two categories, 1 ~ 2 and 3 ~ 4, and then we calculated an odds ratio of replying more negative answer (3 ~ 4).

#### Statistical analysis

Descriptive statistics were used for each category of the following variables: socio-demographic characteristics, occupation, and shift work experience. To examine the association between night shift rotation interval and sleep quality, univariate logistic regression analysis was conducted. Besides the night shift rotation interval, factors such as age [35], body mass index [36], smoking status [37], alcohol consumption [38], exercise [35], being allowed to sleep during a night shift [39], work experience [40], and shift work experience [40] were hypothesized to influence sleep quality of sleep, and thus were adjusted for in the multivariate analyses.

The above categorical variables were converted into dummy variables, and then analyzed using multivariate logistic regression. The associations between each adjusted variable and sleep quality were also analyzed through univariate logistic regression analysis. All statistical tests were performed using PASW 18.0 (IBM SPSS Inc., Chicago, IL, USA). Statistical significance was set at 0.05.

## Results

Of the 2818 total subjects, 431, 1517, and 850 were in groups A, B, and C, respectively. For the age group distribution in each group, groups A and B had a similar distribution (group A: 84.7 % were 20–29 and 15.3 % were 30–39, group B: 89.2 % were 20–29 and 10.8 % were 30–39), but group C had a high proportion of those aged 30–39 years (78.1 % were 20–29 and 21.9 % were 30–39) (*P* < 0.001). For marital status, groups B and C both had a similar value about proportion of married female (14.4 %, 12.9 %), whereas group A had the highest proportion of married women (25.5 %) (*P* < 0.001). For education level, groups A and B, who work in the same company, had a similar proportion of women with a high school or lower level of education (60.1 and 65.1 %, respectively). However, group C had the highest proportion of those with a high school or lower level of education (74.9 %) (*P* < 0.001). Regarding smoking status, group C had a remarkably high percentage of smokers (group A, 4.4 %; group B, 2.9 %;, and group C, 20.5 %). (*P* < 0.001).

Group C had the lowest proportion of non-drinkers (39.9 %) compared to that of group A (50.8 %) or B (52.0 %). However, moderate drinkers accounted for 26.5 % in group A, 27.2 % in group B, and 32.5 % in group C. Heavy drinkers represented 22.7 % in group A, 22.1 % in group B, and 27.6 % in C (*P* < 0.001). There was no significant difference across the three body mass index groups. The percentage of those in the underweight group were 11.8, 13, and 14 % and in the overweight group were 13.5, 14, and 17.6 % for groups A, B, and C, respectively (*P* = 0.062).

Concerning exercise, group A had the highest proportion of those who exercised ≥3 times per week (group A, 63.3 %; group B, 60.3 %; and group C, 54.8 %) (*P* = 0.005). Group A also had the highest proportion of women who were allowed to sleep during night shifts (97.2 %) (group B, 90.0 % and group C, 76.4 %) (*P* < 0.001). Regarding work experience, group B had the greatest proportion of those with <5 years (51.9 %) followed by group C (39.8 %) and A (22.5 %). Group A had the highest proportion of those with ≥10 years of experience (35.5 %), while groups B and C showed similar proportions (23.1 and 25.8 %, respectively) (*P* < 0.001). A similar trend was found for shift work experience. Group B had the highest percentage of those with <5 years of shift work experience (52.8 %), while group A had the highest proportion of those with ≥10 years of shift work experience (35.9 %) (*P* < 0.001). Overall, all of the measured factors except for work experience and shift work experience differed for each company. Furthermore, groups B and C had higher proportions of those with shorter work experience than group A did (Table 1).

**Table 1:** General characteristics of study subjects

Variables	Group A	Group B	Group C	*P*-value^a^
	Number (%)	Number (%)	Number (%)	
Age				<0.001
20’s	365 (84.7)	1,371 (89.2)	664 (78.1)	
30’s	66 (15.3)	166 (10.8)	186 (21.9)	
Marital status				<0.001
Unmarried	320 (74.2)	1,311 (85.3)	737 (86.7)	
Married	110 (25.5)	222 (14.4)	110 (12.9)	
Divorced	1 (00.2)	4 (00.3)	3 (00.4)	
Education Level				<0.001
≤ High school	261 (60.6)	1,000 (65.1)	637 (74.9)	
≥ College	170 (39.4)	537 (34.9)	213 (25.1)	
Smoking				<0.001
No	412 (95.6)	1,492 (97.1)	676 (79.5)	
Current	19 (04.4)	45 (02.9)	174 (20.5)	
Alcohol consumption				<0.001
No	219 (50.8)	799 (52.0)	339 (39.9)	
Mild	114 (26.5)	418 (27.2)	276 (32.5)	
Moderate	98 (22.7)	340 (22.1)	235 (27.6)	
Obesity (BMI)				0.062
18.5–24.9	322 (74.7)	1,122 (73.0)	581 (68.4)	
< 18.5	51 (11.8)	200 (13.0)	119 (14.0)	
≥ 25.0	58 (13.5)	215 (14.0)	150 (17.6)	
Exercise				0.005
< 3 times per weeks	158 (36.7)	610 (39.7)	384 (45.2)	
≥ 3 times per weeks	273 (63.3)	927 (60.3)	466 (54.8)	
Sleep during working				<0.001
No	12 (02.8)	154 (10.0)	431 (23.6)	
Yes	419 (97.2)	1,383 (90.0)	1,537 (76.4)	
Job tenure				<0.001
< 5 years	97 (22.5)	798 (51.9)	338 (39.8)	
5–9 years	181 (42.0)	384 (25.0)	293 (34.5)	
≥ 10 years	153 (35.5)	355 (23.1)	219 (25.8)	
Job tenure (Shift work)				<0.001
< 5 years	100 (23.2)	811 (52.8)	341 (40.1)	
5–9 years	198 (45.9)	425 (27.7)	354 (41.6)	
≥ 10 years	133 (35.9)	301 (19.6)	155 (18.2)	

^a^comparison by chi-squared test

Table 2 shows the relationship between our measured variables including the night shift rotation interval and sleep quality. When we compared the different night shift rotation interval across the three groups, the odds ratios (OR) and 95 % confidence intervals (CI) of having severely low quality of sleep were as follows: group B had an OR of 1.456 (95 % CI 1.171–1.811) and group C had an OR of 2.348 (95 % CI 1.852–2.977) with group A as the reference group. These data suggest that those in group C are most likely to experience a low quality of sleep. For statistical adjustment, factors such as age, body mass index, smoking status, drinking, exercise, being allowed to sleep during night shifts, work experience, and shift work experience were used. After adjustment, group B had an OR of 1.419 (95 % CI 1.134–1.777) and group C had an OR of 2.238 (95 % CI 1.737–2.882) with group A as the reference group.

**Table 2:** Association between quality of sleep and shift work, and other factors

Variables	Crude^a^		Adjusted^b^	
	OR^c^	95 % CI^d^	OR	95 % CI
Length of night–shift Cycle				
Group A (6 weeks)	1		1	
Group B (4 weeks)	1.456	1.171–1.811	1.419	1.134–1.777
Group C (12 days)	2.348	1.852–2.977	2.238	1.737–2.882
Age				
20–29	1		1	
30–39	0.834	0.665–1.046	0.883	0.670–1.163
Obesity (BMI)				
18.5–24.9	1		1	
< 18.5	1.000	0.787–1.270	1.059	0.844–1.329
≥ 25.0	1.119	0.891–1.403	1.072	0.865–1.328
Smoking				
No	1		1	
Current	1.990	1.482–2.670	1.415	1.053–1.899
Alcohol consumption				
No	1		1	
Mild	1.193	0.987–1.443	1.133	0.948–1.354
Moderate	1.611	1.314–1.976	1.418	1.170–1.720
Regular Exercise				
< 3 times per weeks	1		1	
≥ 3 times per weeks	0.962	0.818–1.133	0.936	0.803–1.092
Job tenure				
< 5 years	1		1	
5–9 years	0.904	0.747–1.093	0.552	0.340–0.896
≥ 10 years	0.798	0.653–0.976	0.474	0.275–0.816
Job tenure (Shift work)				
< 5 years	1		1	
5–9 years	0.937	0.781–1.125	1.557	0.964–2.515
≥ 10 years	0.848	0.682–1.053	1.906	1.102–3.296
Sleep during working				
Impossible	1		1	
Possible	1.015	0.810–1.273	1.199	0.951–1.511

^a^analysised by simple logistic regression analysis

^b^adjusted by age, obesity, smoking, alcohol consumption, regular exercise, job tenure, shift work tenure, sleep during working

^c^odds ratio, ^d^confidence interval

In the covariate analysis, sleep quality tended to be better among those aged 30–39 compared to those aged 20–29, but this finding was not statistically significant (OR 0.834, 95 % CI 0.665–1.046). Compared to the normal weight group, both the underweight and overweight groups had a higher percentage of women with low sleep quality, but no statistical significance was found. Compared to non-smokers, smokers were more likely to have low sleep quality (OR 1.415, 95 % CI 1.053–1.899). In addition, alcohol consumers tended to experience low sleep quality, but the relationship was only statistically significant among the heavy drinkers (OR 1.418, 95 % CI 1.170–1.720). The association was not statistically significant among the moderate drinkers (OR 1.133, 95 % CI 0.948–1.354).

Exercise had no effect on sleep quality, but those who exercised ≥3 times per week had a slightly low sleep quality (OR 0.962, 95 % CI 0.818–1.133). Prior to adjustment, only those with ≥10 years of work experience were likely to have a high quality of sleep (OR 0.798, 95 % CI 0.653–0.976). However, after adjustment, the group with 5–10 years of experience was significantly more likely to have higher sleep quality than the group with <5 years work experience was. In general, subjects with longer work experience were more likely to experience a higher quality of sleep.

Concerning shift work experience, longer shift work experience tended to result in a high quality of sleep before adjustment, but after adjustment, longer shift work experience was more likely to lead to a low quality of sleep. In particular, this finding was statistically significant in those with ≥10 years of experience (OR 1.906, 95 % CI 1.102–3.296). Sleep quality was not found to be related with being allowed to sleep during night shift work (OR 1.199, 95 % CI 0.951–1.511).

Table 3 shows the distribution of each group’s PSQI score, their average score, and the average amount of sleep per night. Group C had the highest proportion of women with a low sleep quality (59.8 %, ≥6 points in PSQI) compared to groups A and B (38.7 % and 48.0 %, respectively) (*P* < 0.001). The average PSQI score was also highest in group C (group A, 5.32 ± 2.98; group B, 5.75 ± 2.95; and group C, 6.63 ± 3.01). Moreover, the average amount of sleep was lowest in group C (group A, 7.06 ± 1.58 h; group B, 6.63 ± 1.24 h; and group C, 5.95 ± 1.47 h).

**Table 3:** General PSQI results and mean sleep time comparison by group

Variables	Group A	Group B	Group C	*P*-value^a^
	*N* (%)^b^	*N* (%)	*N* (%)	
Sleep quality				<0.001
Good	264 (61.3)	800 (52.0)	342 (40.2)	
Poor	167 (38.7)	737 (48.0)	508 (59.8)	
Mean PSQI^c^ Score	5.32 ± 2.98	5.75 ± 2.95	6.63 ± 3.01	
Mean sleep time(hour)	7.06 ± 1.58	6.63 ± 1.24	5.95 ± 1.47	

^a^comparison by chi-squared test

^b^Percentage of subjects who reply above 3 at each PSQI questionnaire

^c^Pittsburgh Sleep Quality Index

Table 4 shows the percentage of those who answered above 3 at some PSQI question. Those questions are related with subjective sleep quality, sleep latency (cannot get to sleep within 30 min), sleep disturbance (wake up in the middle of the night or early morning), daytime dysfunction (sleepiness or concentration disturbance). For all three groups, only group C was significantly more likely to not fall asleep within 30 min (OR 1.294, 95 % CI 1.020–1.643). Those with a poor subjective sleep satisfaction in groups A, B, and C were 30.2 %, 41.1 %, and 41.9 %. The OR was highest in group C (OR 1.669, 95 % CI 1.304–2.136), suggesting that group C had the lowest subjective sleep satisfaction of all three groups. In addition, the proportion of those who answered experiencing sleepiness and a difficulty concentrating during day-time shifts was highest in group C, followed by groups B and A. Group C also had the highest OR for sleepiness (OR 2.096, 95 % CI 1.596–2.752) and low concentration (OR 2.757, 95 % CI 1.815–4.187).

**Table 4:** Odds ratio about number of subjects who reply above 3 points at some PSQI questionnaire by group

Variables	Group A	Group B	Group C
	OR^a^ (95 % CI^†^)	OR (95 % CI)	OR (95 % CI)
Q1^b^	Referent	1.617 (1.285–1.304)	1.669 (1.304–2.136)
Q2^c^	Referent	1.131 (0.907–1.410)	1.294 (1.020–1.643)
Q3^d^	Referent	1.176 (0.950–1.457)	1.014 (0.804–1.279)
Q4^e^	Referent	1.325 (1.022–1.718)	2.096 (1.596–2.752)
Q5^f^	Referent	1.920 (1.279–2.884)	2.757 (1.815–4.187)

^a^odds ratio, ^†^confidence interval

^b^During the past month, how would you rate your sleep quality overall? (PSQI 6)

^c^Cannot get to sleep within 30 min (PSQI 5–a)

^d^Wake up in the middle of the night or early morning (PSQI 5–b)

^e^During the past month, how often have you had trouble staying awake while driving, eating meals, or engaging in social activity? (PSQI 8)

^f^During the past month, how much of a problem has it been for you to keep up enough enthusiasm to get things done? (PSQI 9)

## Discussion

Our study focused on the influence of the night shift rotation interval on sleep quality and demonstrated that the group with the shortest night shift rotation interval (group C) had the lowest sleep quality (OR 2.348, 95 % CI 1.852–2.977). This result persisted after adjustment for important covariates (OR 2.238, 95 % CI 1.737–2.882).

Various factors affect shift work. It has been reported that the type of shift work, direction of shift rotation, number of consecutive night shifts, and number of off-duty days during shift work other than a night shift rotation interval can affect sleep quality. In this study, although each group worked a different shift systems (group A, 4-team 3-shift system whereas groups B and C, 3-team 2-shift system), group C was the most likely to have a low quality of sleep, even when compared with group B which has the same 3-team 2-shift system.

In addition, all of these groups worked on a non-consecutive shift system, which has been reported to hardly affect sleep quality [1]. In our study, all three groups have slow rotating shift, the number of consecutive night shifts did not differ greatly between groups (groups A and B worked five days and group C worked four days consecutively). Moreover, longer successive night shifts have been found to be associated with lower sleep quality; however, we were not able to investigate the influence of longer successive night shifts.

In addition, the clockwise rotation of shift work has been reported to lead to better sleep quality [41]. However, group A’s direction of shift rotation was counter-clockwise. In addition, the number of off-duty days was the same (two days) in all three groups. Therefore, any differences in sleep quality in this study were likely due to the night shift rotation interval. The results of this study suggest that a shorter night shift rotation interval leads to lower sleep quality.

Generally, shift workers are known to have a shorter average amount of sleep, daytime sleepiness, and/or low concentration. Avoidance of these consequences of low sleep quality is important because these conditions affect the workers’ cognitive performance and behaviors during work hours, therefore potentially increasing the incidence of occupational injuries [42, 43].

According to Wilkins et al. [44], the average amount of sleep was 6.7 h for fixed night shift workers, 6.3 h for shift workers with a weekly changing schedule, and 5.8 h for shift workers with shorter shift rotation. We also found a shorter night shift rotation interval to be associated with a shorter average amount of sleep (average amount of sleep was 7.06 h for group A, 6.63 h for group B, and 5.95 h for group C), which is congruent with the previous study’s results. In addition, a detailed analysis of the PSQI demonstrated that group C had the worst subjective quality of sleep as well as highest proportion of daytime sleepiness and difficulty maintaining concentration, followed by groups B and A, suggesting that a shorter night shift rotation interval is associated with lower subjective sleep quality and more severe daytime sleepiness.

Previous studies have found older shift workers to have more changes in their circadian rhythms called morningness as well as physical weakness, leading to a lower quality of sleep than their younger counterparts have [31, 35]. However, age was not associated with sleep quality in this study. One reason for this discrepancy might be that, in the previous studies, the age groups with low sleep quality mostly comprised adults older than 40 years, which is outside of the age range included in the present study.

Obesity is known to be associated with sleep apnea as well as to directly affect the quality of sleep [36]. However, in this study, obesity was not significantly related with sleep quality. We think the result caused by that subjectives are more sensitive young women than other age. Furthermore, previous studies have reported that smokers experience a low quality of sleep [37], and our study also found smokers to have a lower quality of sleep than non-smokers (OR 1.415, 95 % CI 1.053–1.899).

Alcohol consumption, like obesity, has been found to be associated with sleep-related diseases including sleep apnea. Although moderate drinking produced conflicting results in its association with sleep, heavy drinking has been reported to be related with a low quality of sleep [38]. In this study, heavy drinkers were more likely to experience a lower quality of sleep than non-drinkers were (OR 1.418, 95 % CI 1.170–1.720). In addition, exercise was not significantly related with sleep quality. However, in the present study, we did not evaluate the time of day the exercise was performed, which is an important factor in evaluating sleep quality [35].

Rosekind et al. [39] performed a study at the US National Aeronautics and Space Administration and found that pilots who took a 40-min cockpit nap during a flight ≥7 h had improved safety and efficiency, whereas those who took a 10–15 min nap had lowered arousal levels. Their study is the only experimental study ever conducted on a cockpit nap. Based on their study, a nap of certain amount is needed in order to be an effective solution to sleepiness during night work. In our study, those who were allowed to sleep during night shifts had a slightly lower occurrence of daytime sleepiness and concentration loss, yet these findings were without statistical significance. Those who were allowed to sleep during night shifts also had a slightly higher quality of sleep than those who were not allowed to sleep during night shifts, but this finding also lacked statistical significance. The reason for this is thought to be that a detailed analysis was not performed because we could not know when and how long the subjects could sleep during night shifts due to the limitations in the collection of study data.

Longer work experience and shift work experience are known to lower the risk for cardiovascular diseases, by helping workers adapt to their environment [40]. However, no study has discovered the association between these variables and sleep quality to date. This study demonstrated that longer work experience (≥10 years of experience) might be associated with high sleep quality (OR 0.474, 95 % CI 0.275–0.816). Nonetheless, a longer experience in shift work was associated with a lower quality of sleep (OR 1.096, 95 % CI 1.102–3.296).

### Limitations

Due to the limitations in the collection of the data, shift changeover times of subjects were not clearly identified because all of the data used in the present study were collected at one employee physical examination. Because the reported quality of sleep may differ depending on whether the subjects were currently working day shifts or night shifts at the time of data collection [41], further studies that assess this factor are needed.

There is a difference on daily working time. Both B, C team work same duration for 12 h, but A team has 8 h working time. It is possible that may affect to sleep quality. But after eliminating A group, we analyzed quality of sleep between B and C group. In the comparison of group that has same daily working time, C group worse quality of sleep than B group (OR 1.440, 95 % CI 1.187–1.747).

## Conclusion

Despite several limitations, this study demonstrated that a shorter night shift rotation interval provides less time to recover a disrupted circadian rhythm, thus resulting in a low reported sleep quality. So in building a shift work schedule, securing enough period of night shift rotation interval has a benefit to maintain and improve the sleep quality of employees. Applying in practice, in Korea, weekly rotation is very frequent. If this rotation changed into a 2 weeks or longer period schedule, we expect that could improve sleep quality of employees. Also, night shift rotation interval is correlated with numbers of rotation team. In same rotation schedule and type (for example 1 weeks, 2-shift), 4 team has longer night shift rotation interval than 3 team. So increasing numbers of team is concluded to better quality of sleep.

There is no doubt the elimination of shift work is most effective to workers’ health management. However shift work is influenced by various factors such as socioeconomic factors. Future research should aim to find the most optimal model of shift work for minimizing human health effect.
