# Erratum to: The relationship between quality of sleep and night shift rotation interval

**DOI:** 10.1186/s40557-016-0090-7

**Published:** 2016-01-25

**Authors:** Jae Youn Kim, Chang Ho Chae, Young Ouk Kim, Jun Seok Son, Ja Hyun Kim, Chan Woo Kim, Hyoung Ouk Park, Jun Ho Lee, Soon Kwon

**Affiliations:** Department of Occupational & Environmental Medicine, School of Medicine, Sungkyunkwan University, Samsung Changwon Hospital, 158, Paryong-ro, Changwon-si, 51353 Changwon, Gyeongsangnam-do Korea (Republic)

## Erratum

It has come to our attention that there is an error in one of the author names in this article [1]. The author name Soon Il Kwon was incorrectly spelt as Sun Il Kwon. The original article has now been corrected. The publisher apologises for any inconvenience caused.
